# Humans combine value learning and hypothesis testing strategically in multi-dimensional probabilistic reward learning

**DOI:** 10.1371/journal.pcbi.1010699

**Published:** 2022-11-23

**Authors:** Mingyu Song, Persis A. Baah, Ming Bo Cai, Yael Niv

**Affiliations:** 1 Princeton Neuroscience Institute, Princeton University, Princeton, New Jersey, United States of America; 2 Department of Psychology, Princeton University, Princeton, New Jersey, United States of America; 3 International Research Center for Neurointelligence (WPI-IRCN), The University of Tokyo, Tokyo, Japan; Dartmouth College, UNITED STATES

## Abstract

Realistic and complex decision tasks often allow for many possible solutions. How do we find the correct one? Introspection suggests a process of trying out solutions one after the other until success. However, such methodical serial testing may be too slow, especially in environments with noisy feedback. Alternatively, the underlying learning process may involve implicit reinforcement learning that learns about many possibilities in parallel. Here we designed a multi-dimensional probabilistic active-learning task tailored to study how people learn to solve such complex problems. Participants configured three-dimensional stimuli by selecting features for each dimension and received probabilistic reward feedback. We manipulated task complexity by changing how many feature dimensions were relevant to maximizing reward, as well as whether this information was provided to the participants. To investigate how participants learn the task, we examined models of serial hypothesis testing, feature-based reinforcement learning, and combinations of the two strategies. Model comparison revealed evidence for hypothesis testing that relies on reinforcement-learning when selecting what hypothesis to test. The extent to which participants engaged in hypothesis testing depended on the instructed task complexity: people tended to serially test hypotheses when instructed that there were fewer relevant dimensions, and relied more on gradual and parallel learning of feature values when the task was more complex. This demonstrates a strategic use of task information to balance the costs and benefits of the two methods of learning.

## Author summary

When solving complex tasks with many potential solutions, we often try the solutions one at a time until success. However, when the set of solutions is too large to exhaust, or if feedback is noisy, we may also rely on implicit reinforcement learning to evaluate multiple options concurrently. In this study, with a novel task that allows participants to actively search for unknown rules in a large search space, we find that human participants combine both strategies, namely serial hypothesis testing and reinforcement learning, in their decisions. Depending on the complexity of the task participants change the balance between the strategies, in line with their costs and benefits.

## Introduction

Learning in a complex environment, with numerous potentially relevant factors and noisy outcomes, can be quite challenging. For example, when learning to make bread, many decisions need to be made: the amount of yeast to use, the flour-to-water ratio, the proof time, the baking temperature. It can be hard to learn the correct decision for each of these factors, especially when the results are variable even if following the same procedure: the ambient temperature may affect rising, the oven temperature may not be as accurate as its marks, etc., making the outcome unreliable.

Learning scenarios like this are quite common in life. In controlled laboratory experiments, each of the key components of such learning—multiple dimensions of features interacting, probabilistic outcomes, and active choice of learning examples—has traditionally been investigated separately. For instance, decisions based on combining multiple factors (features) are common in category learning tasks [1, 2] where multidimensional rules determine the category boundaries. However, feedback is often deterministic in these tasks, making it unclear how multidimensional learning occurs when choice outcomes are less reliable. In contrast, the need to integrate and learn from stochastic feedback has been widely studied in probabilistic learning tasks [3–5], but often with simple rules that involve only one relevant feature dimension. Finally, the freedom to choose learning examples (rather than selecting among a few available options) is at the core of active learning [6–8], where studies have focused on testing how well human decisions accord with principles of information gain maximization [9] or uncertainty-directed exploration [10].

As few tasks have combined all these components (but see [11] for active learning with probabilistic multidimensional stimuli), it remains unclear how people learn actively in an environment with complex rules (with multiple and potentially an unknown number of relevant dimensions) and probabilistic feedback. To study this, we developed a novel decision task: participants were asked to configure three-dimensional stimuli by choosing what features to use in each dimension, earning rewards that were probabilistically determined by features in a subset or all of these dimensions. To earn as much reward as possible, participants needed to figure out which dimensions were important through trial-and-error, and learn what specific features yielded rewarding outcomes in those dimensions.

Despite the computational challenge and combinatorial explosion of possible solutions, human beings are remarkably good at solving such complex tasks. Usually, after a few successful or unsuccessful attempts, an amateur baker will gradually figure out the rules for bread-making. Similarly, participants in our task improved their performance over time, and learned to correctly identify rewarding features through experience. To understand how they achieved this, we turned to the extensive literature regarding algorithms that support learning when it is not clear what features are relevant (i.e., representation learning) [12, 13]. Previous work has suggested several mechanisms for such learning [14, 15]: a value-based reinforcement-learning mechanism that incrementally learns the value of stimuli based on trial-and-error feedback, and a rule-based mechanism that explicitly represents and evaluates hypotheses. In previous studies, the two mechanisms were often examined separately, as which of them is used often depends on the specific task. For instance, in probabilistic reward learning tasks, people have been shown to learn through trial-and-error to identify relevant dimensions, and gradually focus their attention onto the rewarding features in those dimensions [3–5]. In contrast, in category learning, people seem to evaluate the probability of all possible rules via Bayesian inference, with a prior belief favoring simpler rules [2, 16, 17] (note there also exists other strategies in category learning [14, 15, 18, 19], e.g., exemplar-based models). However, the two learning mechanisms are likely simultaneously engaged in most tasks [20], and contribute to different extents depending on how efficient they are in each specific setting. Direct hypothesis-testing can be more efficient when fewer hypotheses are likely and when feedback is relatively deterministic, whereas incremental learning may be more beneficial with numerous possible combinations and stochastic outcomes.

Here, we systematically examined the integration of the two learning mechanisms and how it depends on task condition. Specifically, we varied task complexity by setting the rules such that one, two, or all three dimensions of the stimuli were relevant for obtaining reward; in addition, we manipulated whether such information (i.e., rule dimensionality) was explicitly provided to participants. We fit computational models that represent each learning mechanism, and their combination, to participants’ responses, and compared how well they predicted participants’ choices. We found evidence that people used a combination of the two learning mechanisms when solving our task. Furthermore, when participants were informed of the task complexity, they used this information to set the balance between the two mechanisms, relying more on serial hypothesis testing when the task was simpler, with fewer candidate rules, and more on reinforcement learning when more rules were possible. Our findings shed light on how rule-based and value-based mechanisms cooperate to support representation learning in complex and stochastic scenarios, and suggest that humans use task complexity to evaluate the effectiveness of different learning mechanisms and strategically balance between them.

## Results

### Experiment: The “build your own icon” task

In our task, stimuli were characterized by features in three dimensions: color (red, green, blue), shape (square, circle, triangle) and texture (plaid, dots, waves). In each of a series of games, a subset of the three dimensions was relevant for reward, meaning that one feature in each of these relevant dimensions would render stimuli more rewarding (henceforth the “rewarding feature”).

**Fig 1:**
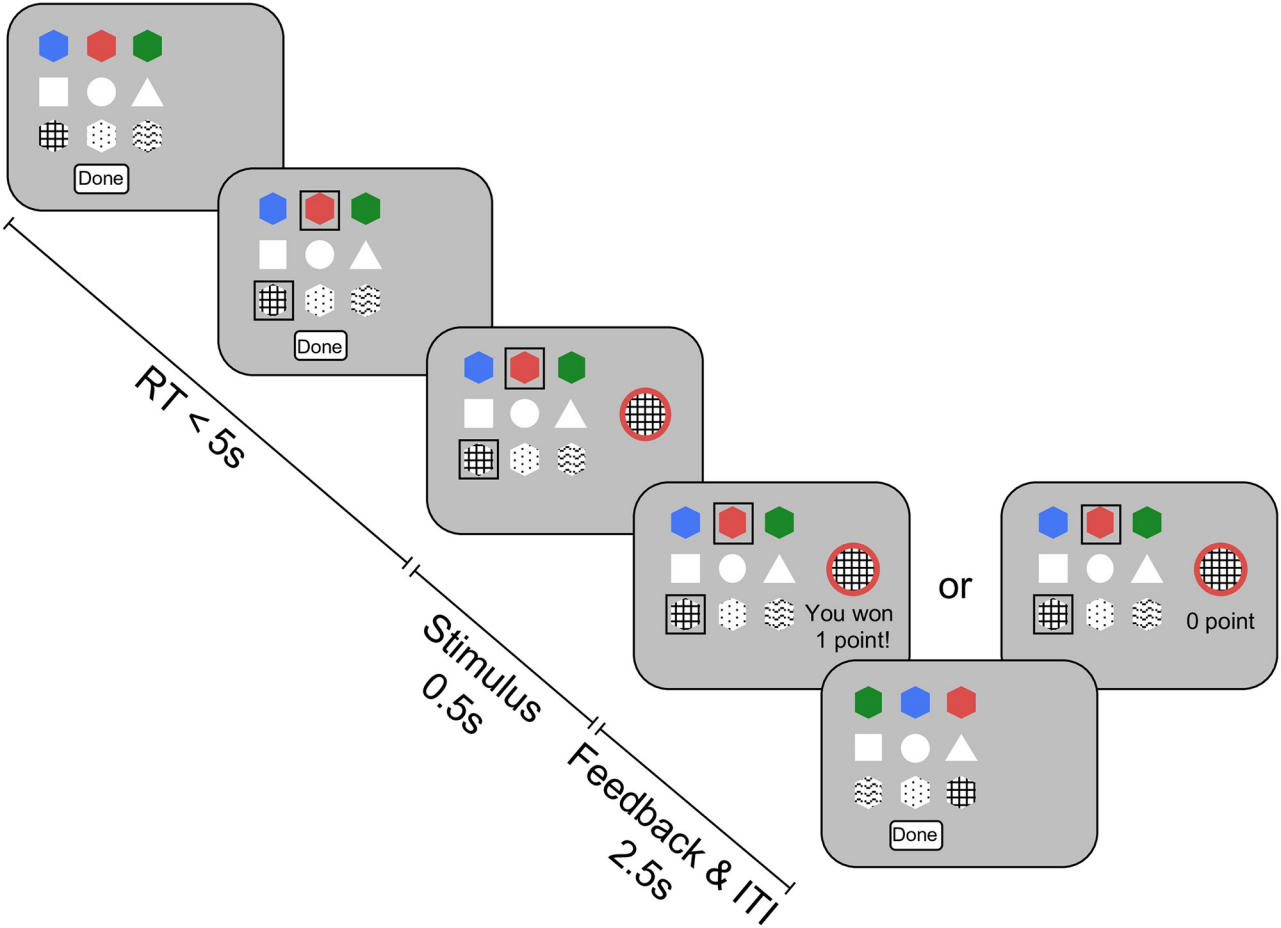
The “build your own icon” task. Participants built stimuli by selecting a feature in zero to three dimensions (marked by black squares). After hitting “Done”, the stimulus showed up on the screen, with features randomly determined for any dimension in which participant did not make a selection (in this example, circle was randomly determined). Reward feedback was then shown.

To earn rewards and figure out the underlying rule, participants were asked to configure stimuli (“icons”) by selecting features for any of the dimensions (Fig 1); for dimensions in which they did not make a selection, the computer would randomly select a feature. The resulting stimulus was then shown on the screen, and the participant would receive probabilistic reward feedback (one or zero points) based on the stimulus: the more rewarding features included in the stimulus, the higher the reward probability, with the lowest reward probability being *p* = 0.2 and the highest being *p* = 0.8 (see Table 1). The participants’ goal was to earn as many reward points as possible.

**Table 1:** The reward probability of a stimulus in each game type (1D, 2D, and 3D-relevant games) was determined by the number of rewarding features in the stimulus. Each row corresponds to one game type. Across all game types, the reward probabilities were 20% if the stimulus contained no rewarding features, 80% if it contained all rewarding features, and linear interpolations between 20% and 80% if it contained a subset of rewarding features. For example, in a 3D-relevant game, if the stimulus contained two of the three rewarding features, the reward probability for that trial would be 60%. These probabilities guarantee that a participant who performs randomly would have 40% probability of obtaining a reward across all game types. This can be seen by calculating, for each game type, the chance of randomly choosing a certain number of rewarding features, multiplied by the corresponding reward probability. Equal chance probability across game types ensured that chance behavior would not be informative about the number of relevant dimensions in unknown games.

Game type	Number of rewarding features			
	0	1	2	3
1D-relevant	20%	80%	–	–
2D-relevant	20%	50%	80%	–
3D-relevant	20%	40%	60%	80%

Each game had one, two, or three relevant dimensions (henceforth 1D-, 2D-, and 3D-relevant conditions). This information was provided to participants in half of the games (“known” condition), with the other half designated as “unknown” games. This resulted in six game types in total. Each participant played three games of each type for a total of 18 games, in a randomized order. Each game was comprised of 30 trials. The relevant dimensions and rewarding features changed between games.

102 participants were recruited through Amazon Mechanical Turk. In an instruction phase, participants were told that each game could have one, two or three dimensions that were important for reward, and were explicitly informed about the reward probabilities in Table 1. They were tested on their understanding of the instructions, and each played three practice games with informed rules (relevant dimensions and rewarding features). The main experiment then commenced. In “known” games, the number of relevant dimensions was informed before the start of the game in the form of a “hint”; participants were, however, never told which dimensions were relevant or which features were more rewarding. The start of “unknown” games was also signaled; however, no hint was provided in these games. At the end of each game, participants were asked to explicitly report, to their best knowledge, the rewarding feature for each dimension, or indicate that this dimensions is irrelevant to reward, as well as their confidence level (0–100) in these judgements. After the experiment, participants received a performance bonus proportional to the points they earned in three randomly selected games.

### Learning performance and choice behavior

**Fig 2:**
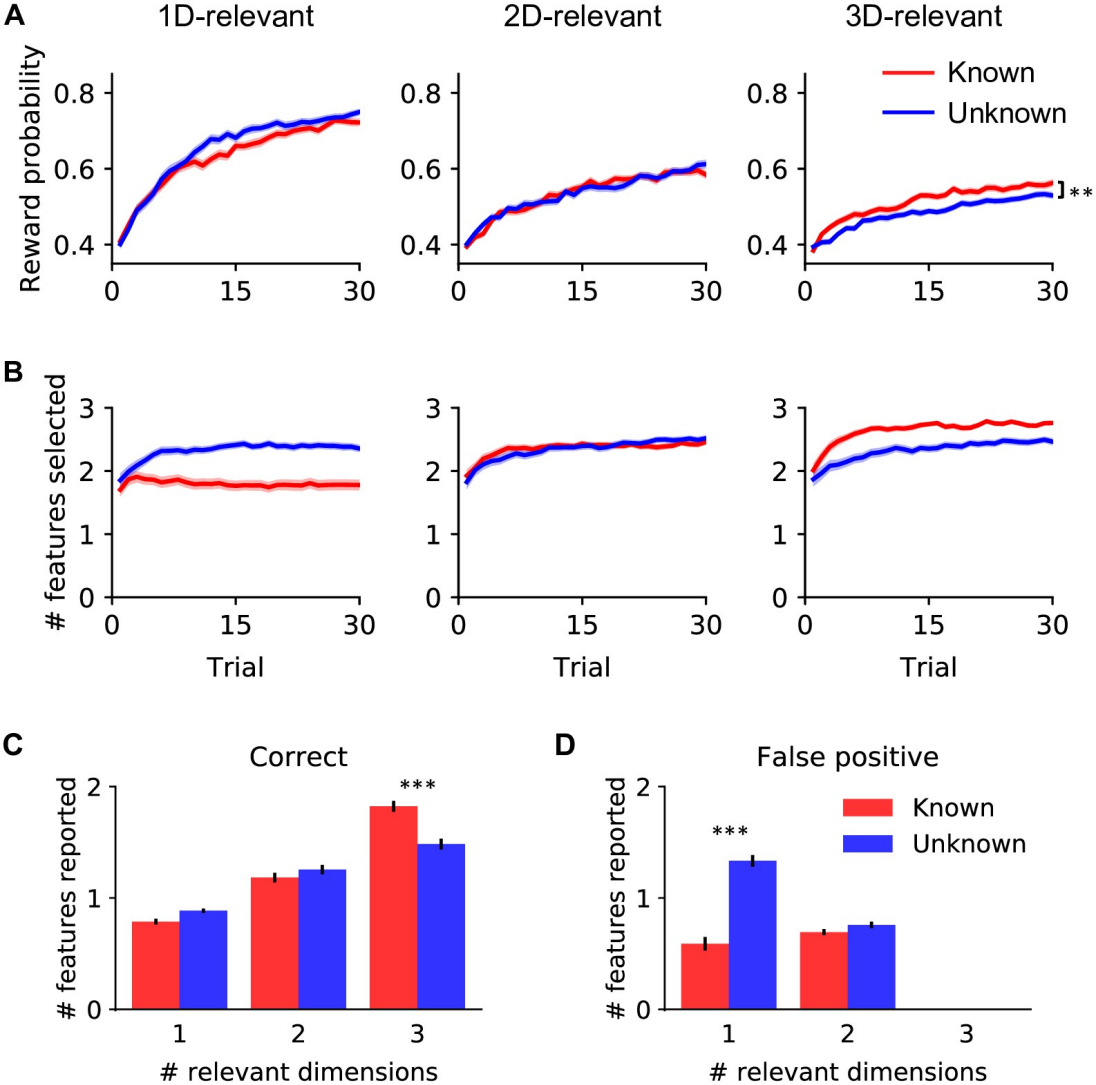
Participants’ behavior in the “build your own icon” task. **(A, B): Performance and choices over the course of a game, by game type**. **(A)** Participants’ average probability of reward (based on the number of rewarding features in their configured stimuli), over the course of 1D-, 2D- and 3D-relevant games (left, middle and right columns). Red and blue curves represent “known” and “unknown” conditions, respectively. For all game types, chance reward probability is 0.4 and 0.8 is the maximum reward probability. Shading (ribbons around the lines) represents ±1 s.e.m. across participants. ** *p* < .01. For grouping of these learning curves by task complexity, see S1 Fig. **(B)** Same as in (A), but for the number of features selected. **(C, D): Responses to post-game questions regarding the rewarding features in each game condition**. **(C)** Average number of correctly-identified rewarding features; **(D)** Average number of false positive responses, i.e., falsely identifying an irrelevant dimension as relevant. *** *p* < .001. Error bars represent ±1 s.e.m. across participants.

Across all six game types, participants’ performance improved over the course of games, with overall better performance and faster learning in less complex games, i.e., games with fewer relevant dimensions (Fig 2A). A mixed-effects regression on reward probability against trial index, task complexity (1D-/2D-/3D-relevant) and game knowledge (known/unknown) showed significant effects of trial index (estimated slope 0.0012 ± 0.0008, *p* < .001) and task complexity (estimated slope −0.044 ± 0.007, *p* < .001), as well as a two-way interaction between trial index and task complexity (estimated slope −0.0027 ± 0.0003, *p* < .001).

The overall worse performance in more complex games was not necessarily a failure of learning, but rather the result of limited experience (only 30 trials per game), as participants’ average reward rate across all games was 90.2% of that of an approximately optimal agent (see Methods) playing this same task (87%, 89% and 95% in the 1D-, 2D- and 3D-relevant games, respectively). Participants’ performance was better when informed of the task complexity in 3D-relevant games (paired-sample t-test on reward probability for 3D-relevant games between “known” and “unknown” conditions: *t*_101_ = 3.37, *p* = .001, uncorrected, same for tests below). There was no effect of game knowledge on performance in simpler games (1D-relevant: *t*_101_ = −1.9, *p* = .060; 2D-relevant: *t*_101_ = 0.02, *p* = .98).

Participants also showed distinct choice behavior in different game types (Fig 2B): a mixed-effects regression on the number of features selected showed significant effects of trial index (more features were selected over time; estimated slope 0.0087 ± 0.0003, *p* = .013) and game knowledge (more features were selected in “unknown” games; estimated slope −0.63 ± 0.09, *p* < .001), two-way interaction effects for all pairs of variables (all *p* < .05), and a significant three-way interaction (*p* < .001). Specifically, in “known” games, participants selected more features when informed that more dimensions were relevant (mixed-effects linear regression slope: 0.29 ± 0.03, *p* < .001); in “unknown” games, unsurprisingly, the number of selected features did not differ between task complexities (*p* = .47).

Participants’ responses to the post-game questions also reflected similar behavioral patterns (see full results in S1(C) Fig). Specifically, we analyzed how often they correctly identified the rewarding features (Fig 2C), and when they falsely identified an irrelevant dimension as relevant (“false positive”, Fig 2D; note that in 3D-relevant games, this measure was 0 by design, thus these games were excluded from this analysis). A two-way repeated-measures ANOVA on correct responses showed a significant main effect of task complexity (*F*_2,202_ = 273.7, *p* < .001), and a significant interaction between task complexity and game knowledge (*F*_2,202_ = 21.3, *p* < .001); the ANOVA on false positive responses showed significant main effects of both task complexity (*F*_1,101_ = 32.0, *p* < .001) and game knowledge (*F*_1,101_ = 93.3, *p* < .001), and a significant interaction between them (*F*_1,101_ = 90.8, *p* < .001). Comparing the “known” and “unknown” conditions: in 1D-relevant games, participants? correct responses did not differ based on condition (Fig 2C; post hoc Tukey test: *t*_101_ = 1.81, *p* = .46), consistent with the choice behavior in Fig 2A; however, participants made more false positive responses in the “unknown” condition (Fig 2D; *t*_101_ = −6.27, *p* < .001), indicating that not knowing the dimensionality of the underlying rule led them to incorrectly attribute rewards to features on multiple dimensions, which might be the reason for the larger number of features selected in the “unknown” condition (Fig 2B). In 3D-relevant games, participants identified more correct features in the “known” condition than in the “unknown” condition (Fig 2C; *t*_101_ = 13.53, *p* < .001), consistent with their better learning performance in “known” 3D-relevant games observed in Fig 2A.

In sum, participants’ behavior was sensitive to both task complexity and game knowledge. They performed better and learned faster in simpler games. Game knowledge had a smaller impact on performance, and participants showed different choice behavior in “known” versus “unknown” games: in “known” games, the number of features they selected was moderated by the instructed task complexity; while in “unknown” games, the number was similar across different complexities.

### Modeling two learning mechanisms

To characterize participants’ learning strategy and explain the behavioral differences between game conditions, we considered two candidate learning mechanisms [15, 20]: an incremental value-based mechanism that learns the value of stimuli based on trial-and-error feedback, and a rule-based mechanism that explicitly represents possible rules and evaluates them. We tested computational models representing each of these mechanisms, as well as a hybrid combination of the two, by fitting each model to participants’ trial-by-trial choices and comparing how well they predict task behavior. We describe each model below; the mathematical details are provided in Methods.

The value-based mechanism was captured by a feature-based reinforcement learning model [3]. Reinforcement learning is commonly used to model behavior in probabilistic reward-learning tasks, where participants need to accumulate evidence across multiple trials to estimate the value of each choice. In particular, we used the **feature RL with decay model** from prior work with a task similar to ours [3]. This model assumes that participants learn values for each of the nine features using a Rescorla-Wagner update rule [21]: feature values in the current stimulus are updated proportional to the reward prediction error (the difference between the outcome and the expected reward). The expected reward for each choice (i.e., combination of features selected) is calculated as the sum of its feature values. At decision time, choice probability is determined by comparing the expected reward for all choices using a softmax function. Additionally, values of features not present in the current stimulus are decayed towards zero. This is particularly relevant for features that had been valued previously but are later not consistently selected, i.e. features that the participant presumably no longer deems to have high values, or those originally selected by the computer. The decay mechanism allows their value to decay down to zero despite not being chosen (otherwise, the model updates only the values of chosen features). Note that, this feature-based RL model, although simple, is well suited to the additive reward structure of the task, and provides a better fit than more complex RL models, such a conjunction-based RL model [22] or an Expert RL model that combines a few RL “experts” each learning different combinations of the dimensions [23].

In contrast to the value-based mechanism, the rule-based mechanism directly evaluates hypotheses regarding what combinations of features yield the most reward in a game, which we refer to as “rules”. In “known” games, there are 9, 27 and 27 possible rules for 1D-, 2D- and 3D-relevant games, respectively; in “unknown” games, all 63 rules are possible.

There are multiple possibilities for how people learn the correct rule. One is to use Bayesian principles to evaluate the probability that each rule is the correct one; we term this a **Bayesian rule-learning model**. After each outcome, this model optimally utilizes feedback to calculate the likelihood of each candidate rule, and combines this with the prior belief of the probability that each rule is correct (initially assumed to be uniform across all rules that accord with the “hint”) to obtain the posterior probabilities of each rule. The expected reward for a choice is then calculated by marginalizing over the posterior belief of all possible rules. Mirroring the reinforcement learning model above, in our implementation, the final choice probability was determined by a softmax function over the expected reward from each choice. In a multi-dimensional category learning task, a similar Bayesian rule learning model has been shown to characterize how people learn categories better than reinforcement learning models [2].

**Fig 3:**
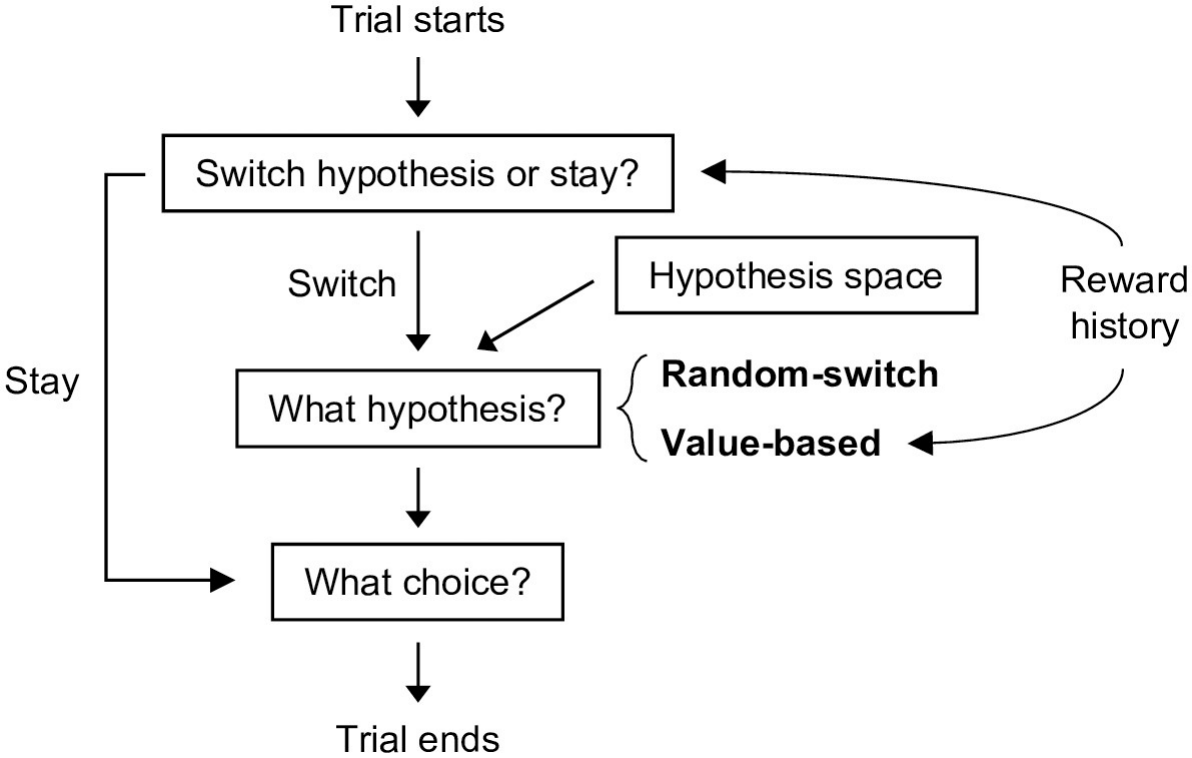
A diagram of the serial hypothesis testing models.

Bayesian inference is computationally expensive and memory-intensive. A simpler alternative for the rule-base strategy is serial hypothesis testing, which assumes that people only test one rule at a time: if the evidence supports their hypothesis, they will continue with it; otherwise, they switch to a different rule, until the correct one is found. The idea of serial hypothesis testing has long roots in the category learning literature [24, 25]. Recently, it has also been applied in probabilistic reward learning tasks [26] and was shown to better account for human behavior than the Bayesian model. Following [26], we considered a **random-switch serial hypothesis-testing model** (random-switch SHT model; Fig 3) that assumes that people test hypotheses about the underlying rule one at a time. When testing a hypothesis, the model estimates its reward probability by counting how often recent choices following this rule were rewarded. The probability of abandoning the current hypothesis and switching to testing a random different hypothesis is inversely proportional to the reward probability. We assumed that people’s choices were often consistent with their hypotheses, but lapsed to random choices with a small (*p* = λ) probability.

The SHT and RL mechanisms are not necessarily mutually exclusive. We thus also considered a hybrid model by incorporating RL-acquired feature values into the choice of a new hypothesis in the serial hypothesis testing model. In particular, when switching hypotheses, the hybrid model favored hypotheses that contain recently rewarded features. We term this model **value-based serial hypothesis testing model** (value-based SHT model; Fig 3; see Methods for detailed equations for all models).

### Evidence for a hybrid learning mechanism

**Fig 4:**
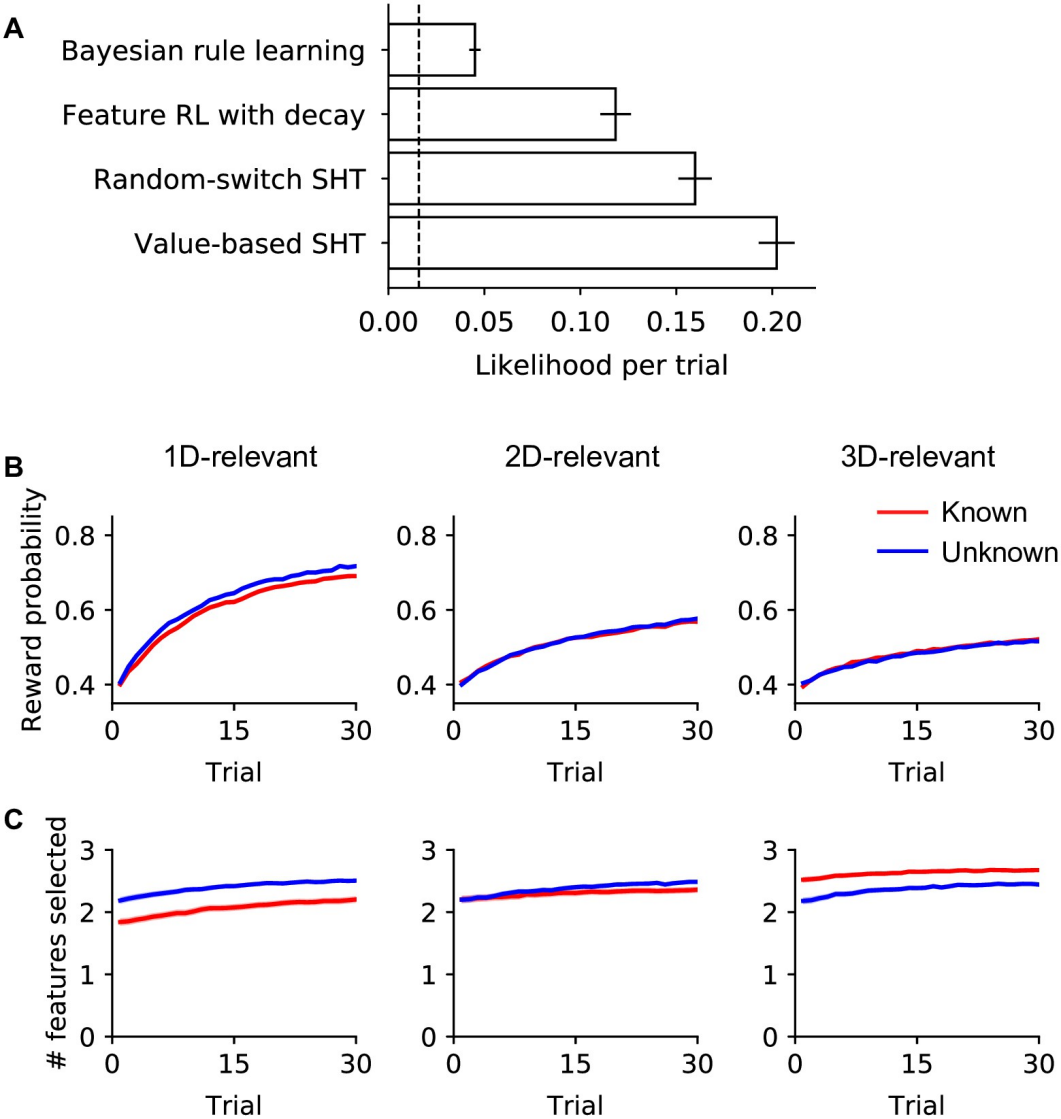
Model comparison supports both reinforcement learning (RL) and serial hypothesis testing (SHT) strategies. **(A)** Geometric average likelihood per trial for each model (i.e., average total log likelihood divided by number of trials and exponentiated). Higher values indicate better model fits. Dashed lines indicate chance. Error bars represent ±1 s.e.m. across participants. **(B, C)** Simulation of the best-fitting value-based SHT model. The same learning curves as in Fig 2 but for model simulation.

We fit all four models to participants’ choice data in this task and evaluated model fits using leave-one-game-out cross-validation (Fig 4A and S2(A) Fig). Among them, the Bayesian rule learning model, even though optimal in utilizing feedback information, showed the worst fit to participants’ choices (likelihood per trial: 0.045 ± 0.003; mean ± s.e.m.). This was potentially because the large hypothesis space (up to 63 hypotheses) made exact Bayesian inference intractable. Both the feature RL with decay model and the random-switch SHT model showed better fits (likelihood per trial: 0.118 ± 0.008 and 0.160 ± 0.009, respectively). Compared to the Bayesian model, both models have lower computation and memory load: the RL model learns nine feature values individually and later combines them; the random-switch SHT model limits the consideration of hypotheses to one at a time. The hybrid value-based SHT model fit the data best (better than either component model; likelihood per trial: 0.202 ± 0.009), suggesting that participants used both learning strategies when solving this task.

There was additional evidence for the involvement of both learning mechanisms in participants’ behavior. The rule-based mechanism was evident from the influence of task instructions: both the numbers of features selected (Fig 2B) and the reported rewarding features in the post-game questions (Fig 2C and 2D) differed between “known” and “unknown” conditions. There is no direct way to incorporate such influences in a reinforcement learning model, but a rule-learning model can easily do so, for instance, by constraining the hypothesis spaces according to the instructions (S3 Fig: the number of features selected differs between known and unknown games for SHT models but not the RL model). In fact, participants adapted their prior beliefs based on their knowledge of the game types (S2(B) Fig): in known games, they assigned a higher prior probability to the hypotheses that are consistent with the task instructions; in unknown games, they deemed more complex rules more likely *a priori*. On the other hand, the influence of value-based learning was evident in the order in which participants clicked on features to make selections. In most cases, participants followed the spatial order in which dimensions appeared on the screen, either top-to-bottom or the reverse. When the clicks violated the spatial orders, however, they followed the order of learned feature values, starting from the most valuable feature, at a frequency significantly above chance (*t*_101_ = 7.63, *p* < .001). Such behavior of following the order of learned feature values instead of the spatial order was more frequent in trials when participants switched hypotheses than when they continued testing the same hypothesis (*t*_101_ = 5.71, *p* < .001; in this analysis, for simplicity, switch trials were identified based on changes in choice), further supporting the value-based SHT model.

In sum, participants’ strategies in this task could not be explained by either reinforcement learning or serial hypothesis testing strategies alone. The combined hybrid model explained participants’ behavior best, also capturing the dependence of performance on task complexity (Fig 4B) and the qualitative differences between choice curves in “known” and “unknown” conditions (Fig 4C), which neither component model could capture (S3 Fig).

### The contribution of the two mechanisms depends on task complexity

Given evidence that participants used both learning strategies in this task, we next asked to what extent each strategy contributed to decision making. We addressed this question by comparing the hybrid model with the two component models: the difference in likelihood per trial between the hybrid model and each component model was taken as a proxy for the contribution of the mechanism not included in the component model. Note that we can treat the RL and SHT models as component models. This is because setting the learning rate to zero effectively “turns off” the RL process, reducing the hybrid model to the random-switch SHT model. Similarly, setting model parameters such that hypotheses are switched every trial “turns off” the SHT process, resulting in a model very similar to the feature RL model (the only difference is the likelihood of returning to the previous hypothesis or choice).

**Fig 5:**
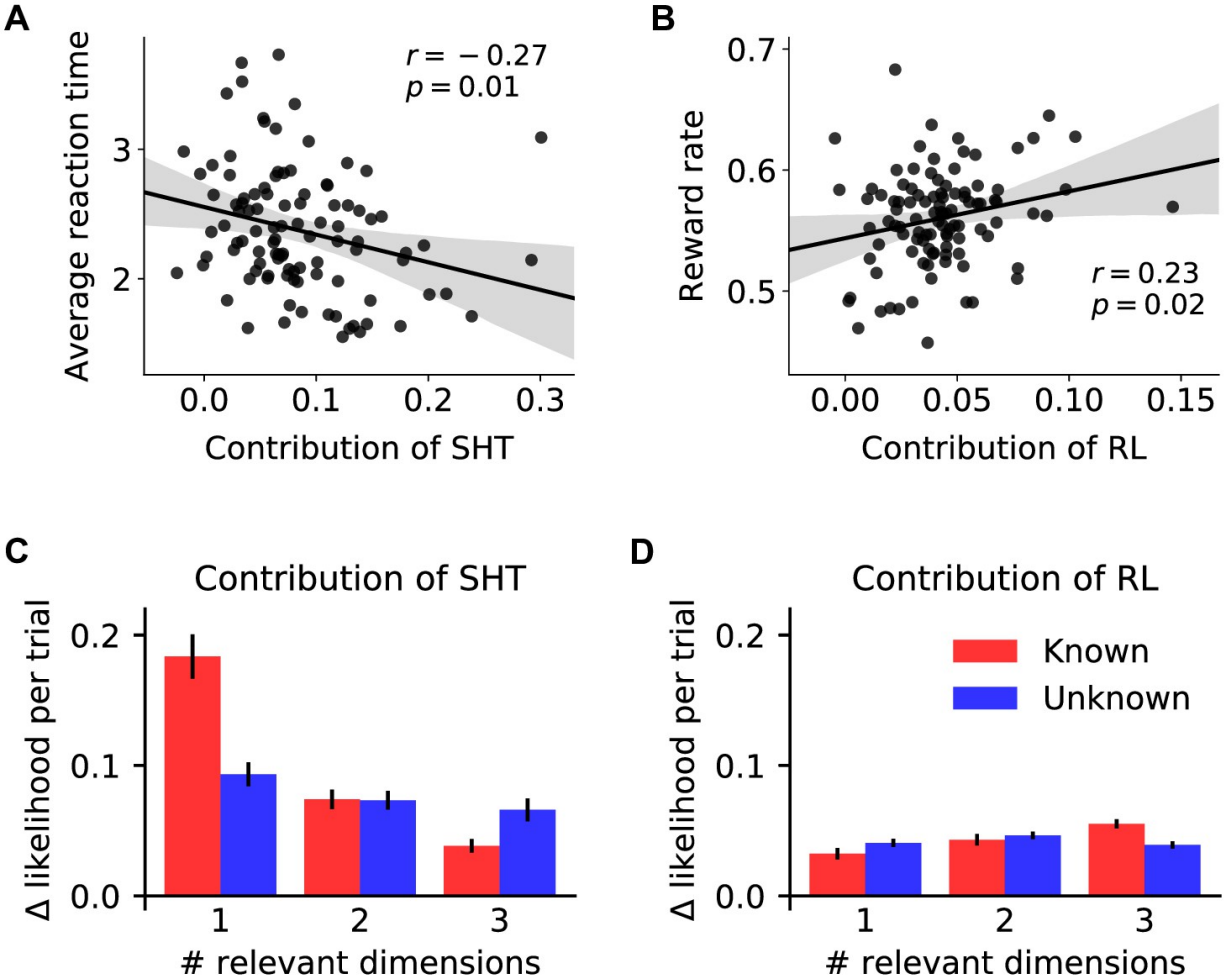
Strategic balance of two learning mechanisms. **(A)** The contribution of serial hypothesis testing (SHT) was inversely correlated with reaction time such that participants who responded faster used SHT to a greater extent. **(B)** The contribution of reinforcement learning (RL) was correlated with average reward rate: participants for whom adding the RL component improved the model fit to a greater extent earned more rewards on the task, on average. Each dot represents one participant. **(C, D)** Contribution of RL and SHT for each game type. The contribution of each component was measured as the difference in likelihood per trial between the hybrid value-based SHT model and the other component model (SHT: the feature RL with decay model; RL: the random-switch SHT model). Error bars represent ±1 s.e.m. across participants.

Across participants, a higher contribution of SHT was associated with a faster reaction time (Fig 5A; Pearson correlation: *r* = −0.27, *p* = .01), and a higher contribution of RL was associated with a higher reward rate (Fig 5B; *r* = 0.23, *p* = .02). These results suggest that, comparatively, serial hypothesis testing was an overall faster and less effortful strategy, and augmenting hypothesis testing with values yielded more reward.

To optimize for reward and reduce mental effort costs, it is advantageous to rely on the serial hypothesis testing strategy when the task is simpler, for instance, in lower-dimensional games with smaller hypothesis spaces. Indeed, when tested separately, the correlation between reward rate and contribution of RL was only significant for 2D- and 3D-relevant games (1D: *r* = −0.03, *p* = .75; 2D: *r* = 0.27, *p* < .01; 3D: *r* = 0.32, *p* < .01; these correlations were significantly different between 2D- and 3D-relevant games and 1D-relevant games [27]: *z* = −2.3, *p* = .023 for 2D vs 1D games, and *z* = −2.7, *p* = .007 for 3D vs 1D games). In contrast, with a larger hypothesis space, serial hypothesis testing is less efficient, and there should be a higher incentive to use the value learning strategy.

We indeed observed such a strategic trade-off between the two learning mechanisms: in “known” games, the contribution of hypothesis testing decreased as the dimensionality of the task increased (Fig 5C; estimated slope in a mixed-effect linear regression: −0.0631 ± 0.0051, *p* < .001), whereas the contribution of value learning increased with task complexity (Fig 5D; estimated slope: 0.0178 ± 0.0013, *p* < .001). In contrast, in “unknown” games, in which task complexity information was unavailable to participants, the contribution of the two mechanisms was more stable across game conditions (estimated slopes: −0.0144 ± 0.0042 for SHT, *p* < .001; −0.0011 ± 0.0012 for RL, *p* = .389, consistent with a significant three-way interaction between task complexity, game knowledge and model component in a repeated measures ANOVA on likelihood difference per trial in Fig 5C and 5D: *F*(2, 202) = 47.9, *p* < .001). Taken together, these results suggest that participants took advantage of information regarding task complexity to strategically balance the use of two complementary learning mechanisms.

## Discussion

Using a novel “build your own icon” task, we studied learning of multi-dimensional rules with probabilistic feedback as a proxy for real-world learning in situations where it is unknown *a priori* what aspects of the task are relevant to solving it, and where learners have agency to intervene on the environment and test hypotheses. In our task, participants created stimuli and tried to earn more rewards by identifying the most rewarding stimulus features. Participants performed this task at various signalled or unsignalled complexity levels (i.e., rewarding features were in one, two or three stimulus dimensions). They demonstrated learning in all conditions, with their performance and strategies influenced by task condition. Through behavioral analyses and computational modeling, we investigated the use of two distinct but complementary learning mechanisms: serial hypothesis testing that evaluates one possible rule at a time and is therefore simple and fast to use, but results in slow learning when many rules are possible and must be tested sequentially, and reinforcement learning that learns about all features in parallel and is more accurate in the long run, but requires maintaining and updating more information. We found that a hybrid model that incorporated the advantages of both mechanisms explained participants’ behavior best. In addition, we showed that human participants demonstrated a strategic balance between the two mechanisms depending on task complexity, suggesting that they were able to gauge which mechanism is more suitable in each condition. Specifically, they tended to use the simpler and faster serial hypothesis testing strategy when they knew that fewer dimensions matter in the decision, but relied more on incrementally learning feature values when they knew multiple dimensions were important.

The current study ties together large bodies of work on reward learning and category learning in multi-dimensional environments. Previous studies have extensively investigated how humans learn about complex but deterministic categorization rules [1, 2, 15], as well as how they learn through trial-and-error to identify a single relevant dimension [3, 22, 28, 29]. The former type of tasks are hard to learn because of the unknown form of the underlying rules, while the latter tasks focus on how humans integrate information over time in stochastic environments. Both are common challenges for human decision-making, and they often co-occur in daily tasks—in new situations, we often do not know *a priori* what aspects of the task are relevant to its correct solution, and feedback may be stochastic due to inherent task properties or—even in deterministic tasks—not knowing what dimensions are relevant to outcomes, making outcomes seem stochastic. Therefore, we imposed both challenges to investigate human learning strategies under such realistically complex scenarios. Our results help unite the various findings on value-based or rule-based strategies in previous studies. We show that learning in complex and stochastic environments engages both strategies, with participants combining them flexibly according to the demands of the task. This can potentially explain why value-based strategies are often observed in probabilistic learning tasks [3–5], and rule-based strategies in category learning tasks [2].

A few studies have pursued a similar path. For example, Choung and colleagues [30] studied a similar probabilistic reward-learning task with multiple relevant dimensions. They examined hypothesis-testing strategies based on values learned with naïve RL models. Through model comparison, they showed that values learned alongside hypothesis testing were carried over when hypotheses switched, consistent with our value-based SHT model. The novelty of our work is in systematically manipulating the complexity of the environment and participants’ knowledge about it, to help provide a comprehensive understanding on how people’s learning strategy adapts to different situations. Another similar set of tasks are contextual bandit problems [31–33], where the amount of reward for each bandit (option) is determined by the context (thus leading to multi-dimensional rules that depend on both stimulus and context). In these tasks, participants were found to use a Gaussian process learning strategy to generalize previous experience to similar instances. Gaussian processes define a probabilistic distribution over the underlying rules, from which one can sample candidate rules as hypotheses. For example, in a task with binary contextual features [31], participants were shown to consider alternative options that were expected to lead to improvements upon the current one, consistent with the rule-based strategy discovered in the current task.

Still, we considered only a simple linear combination of multiple dimensions to determine reward: each relevant dimension contributed equally to reward probability, in an additive manner. In everyday tasks, the composition can be more complex, with different dimensions contributing differently to rewards [11, 29] and potential interactions between dimensions. We postulate that similar hybrid strategies will be adopted regardless. However, it can be hard to model the hypothesis-testing strategy in such scenarios, due to the much larger hypothesis space. An important question is how people construct their hypothesis space, and how likely they deem each hypothesis *a priori*. There is evidence that people favor simpler hypotheses [16]. They also may not have a fixed hypothesis space, but instead construct new hypotheses only when the existing ones can no longer account for observations [34], or they may modify their existing hypotheses on the go with small changes [35].

It is worth noting the unique free-configuration design of the current task. In most representation-learning tasks, stimuli (i.e., the combination of features) are pre-determined, and participants are asked to select between several available options, or make category judgements. These tasks are easy to perform, but it is hard to isolate participants’ preference for single features. Our task directly probed people’s preference (or lack thereof) in each of the three dimensions. In addition, we were able to hold baseline reward probability constant across different game types (participants responding randomly would always earn reward with *p* = 0.4) while varying the complexity of underlying rules, which avoided providing information on rule complexity in “unknown” games. Our free-configuration task also resembles many daily life decisions where choices across multiple dimensions have to be made voluntarily, from ordering a pizza takeout, to planning a weekend getaway trip.

Along with these advantages, the active-learning free-configuration design may also alter the strategy people use, compared to a passive learning scenario. On the one hand, free-choice may encourage hypothesis testing, making this strategy more efficient by allowing participants to seek direct evidence on their hypotheses. On the other hand, learning may be hindered due to confirmation bias, commonly observed in self-directed rule-learning tasks (aka “positive test strategy” [36]). Indeed, participants over-estimated the number of rewarding features in 1D “unknown” games as compared to “known games” (Fig 2D), suggesting that they failed to prune their hypotheses when the underlying rule was simpler. To fully understand the impact of free choice, future work can compare active and passive settings with a “yoked” design. This can help understand whether the findings reported here can be generalized to passive-learning tasks, and what may be unique to the active-learning setting.

To model the integration of the two learning strategies, we introduced the hybrid value-based SHT model. The assumptions in this model are relatively minimal, which can be a reason why the hybrid model failed to quantitatively predict the number of features participants selected (Fig 4C). To improve model prediction, we explored several alternatives for the model’s assumptions (S4 Fig; see Methods for details): (1) not always testing a hypothesis: if none of the hypotheses has a high value, the participant can decide not to test a hypothesis, and let the computer configure a completely random stimulus instead; (2) flexible threshold for determining whether to switch hypothesis or not, based on reward probability of the corresponding game condition (Table 1); (3) favoring choices that are supersets of the current hypothesis: rather than designing stimuli consistent with the current hypothesis (with a lapse rate), participants may tend to select more features than what their hypothesis specifies. The first and third alternative assumptions improved model fits, but the second did not. We then considered a “full” model that used the better alternative for each assumption. This more complex model improved average likelihood per trial on held-out games by 0.033 ± 0.006. In terms of predicting the number of features selected by participants, however, this model behaved similarly to the original hybrid model (S3 Fig). For simplicity, we therefore reported the original hybrid model in the Results. We note that, despite the additional assumptions, the full model predictions still deviated from human behavior, e.g., it under-predicted the differences in the number of selected features between the “known” and “unknown” conditions, compared to the empirical data. This may be due to the simplified assumptions on hypothesis testing: for example, in the model, only one hypothesis was tested at each point in time, and hypothesis switching was purely based on values rather than systematically sweeping through features in a dimension, or decreasing the number of features chosen.

The flexibility of the value-based SHT model opens up the space for exploring more complex hypothesis-testing strategies. For instance, hypotheses may be formed in a hierarchical manner when the rule complexity is unknown, i.e., participants may first reason about the dimensionality of the game, and then the exact rule. Currently, the hypothesis-switching policy depends only on values, whereas participants may start from simpler rules, and switch to more complex rules, as suggested in the SUSTAIN model [37], or vice versa, starting with complex rules and then pruning them to only the necessary components. Another possibility is models that test multiple hypotheses in parallel. In the current model, only one hypothesis is tested at a time, yet participants may consider multiple possibilities simultaneously, for instance, the current configuration and all its subsets. Further, the current study did not evaluate the role of uncertainty-directed exploration [10] and when to terminate it during learning. This is due to the large number of options available in the current task, making the optimal uncertainty-directed policy intractable. Future studies can design targeted tasks to investigate this question. Lastly, the current model assumes that learning of feature values happens in parallel to and independently of hypothesis-testing. However, value learning may also be affected by hypothesis testing. For example, the amount of value update can be gated by the current hypothesis [20, 38]. The current modeling framework (and openly accessible data) can be used in future work to systematically examine these and other alternative models.

In conclusion, we studied human active learning in complex and stochastic environments, with a novel self-configuration decision task. Through behavioral analyses and computational model comparison, our study revealed the strategic integration of two complementary learning mechanisms: serial hypothesis testing using reinforcement-learning values to select new hypotheses. Rule-based and gradual learning systems are often considered opponents or alternatives, whereas our results suggest cooperation rather than arbitration. This may be a general rule in complex, realistic decision tasks. When the going gets rough, the brain would do best to optimally integrate all the methods at its disposal.

## Methods

### Ethics statement

This study was approved by the Institutional Review Board at Princeton University (record number 11968). Formal written consent was obtained from each participant before they started the experiment.

### Experimental procedure and participant exclusion criteria

Participants were recruited online from Amazon Mechanical Turk. They received a base payment of $12 for completing the task, with a performance-based bonus of $0.15 per reward point earned in three randomly-chosen games (one for each task complexity).

Participants went through a comprehensive instruction phase before starting the main task. During the instruction, they were first introduced to the “icons”, and asked to build a few examples. They were then explained the general rules of the experiment, including the complexity levels and their respective reward probabilities (as in Table 1). Participants were tested about these rules and probabilities with a set of multiple-choice questions. For each task complexity level, they were given an example rule, and asked about the reward probability of a few stimuli to test their understanding. Participants had to answer all questions correctly within a fixed number of attempts (5 for questions on the general rules, and 3 for all the other tests). In addition, they played a practice game in each complexity level with the rules informed (including what dimensions were relevant and what features were more rewarding; this information was not available in the main task, even in “known” games, where only the number of relevant dimensions was informed, see details below). During the experimental games, participants were required to respond within 5 seconds on each trial. Participants who did not pass the comprehension tests or missed five consecutive trials at any time in the experiment were not permitted to continue the experiment.

The main task consisted of 18 experimental games. Among them, half were “known” games, in which participants were informed of the number of relevant dimensions (1, 2 or 3) before the game started; the other half were “unknown” games. This corresponded to six game types in total. Each participant played three games of each type in a randomized order. Each game was comprised of 30 trials.

At the end of each game, participants were asked to report the rewarding feature for each dimension through a multiple choice question, or indicate that this dimensions was irrelevant to reward. They were also asked to rate their confidence level (0–100) in these judgements.

106 participants completed the entire experiment, out of which 4 were excluded from our analyses due to poor performance (an overall reward probability less than 0.468, which was two standard deviation below the group average).

### Approximately optimal agent

It is computationally intractable to solve the optimal policy for this task. Therefore we trained a deep Q-network (DQN) [39] on the task to approximate the optimal solution, and compared participants’ performance with this well-trained DQN agent. Specifically, this DQN model uses Bayes rule to update belief states, and deep RL to learn (or approximate) the optimal decision policy.

### Computational models of human behavior

#### Feature-based reinforcement learning with decay model

The feature RL with decay model maintains values (*V*) for each of the nine features (denoted by *f*_*i*,*j*_; *i* and *j* are indices for dimensions and features respectively). At decision time, the expected reward (*ER*) for each possible stimulus configuration *c* is calculated as the sum of its feature values:

$$\begin{array}{c} ER\left(c\right)=\sum_{i}V\left(f_{i,c^{i}}\right), \end{array}$$ (1)

where *c*^*i*^ denotes the feature on dimension *i* of configuration *c*. For dimensions that are unspecified in the configuration (i.e., those the computer will choose randomly), the model uses the average value of all three features.

The choice probability is determined based on *ER*(*c*) using a softmax function, with *β* as a free parameter:

$$\begin{array}{c} P\left(c\right)=\frac{e^{\beta \cdot ER(c)}}{\sum_{c^{\prime }}e^{\beta \cdot ER(c^{\prime })}}. \end{array}$$ (2)

Feature values are updated according to a Rescorla-Wagner update rule, with separate learning rates for features that were selected by the participant (*η* = *η*_*s*_) and those that were randomly determined (*η* = *η*_*r*_). Values of features not in the current stimulus *s*_*t*_ are decayed towards zero with a factor *d* ∈ [0, 1]. *η*_*s*_, *η*_*r*_ and *d* are free parameters.

$$\begin{array}{c} V_{t}\left(f_{i,j}\right)=\{\begin{array}{c} V_{t-1}\left(f_{i,j}\right)+\eta \left(r_{t}-ER\left(c_{t}\right)\right),\;\text{if}\;j=s_{t}^{i}\\ d\cdot V_{t-1}\left(f_{i,j}\right),\;\text{if}\;j\neq s_{t}^{i} \end{array} \end{array}$$ (3)

where *r*_*t*_ is the reward outcome (0 or 1) on trial *t*, and $s_{t}^{i}$ indicates the feature on dimension *i* of *s*_*t*_.

#### Bayesian rule learning model

The Bayesian rule-learning model maintains a probabilistic belief distribution over all possible hypotheses (denoted by *h*). Note that the set of possible hypotheses (the hypothesis space) depends on the current task complexity: in known games, there are 9, 27 and 27 possible hypotheses in 1D, 2D and 3D games, respectively; in unknown games, all 63 hypotheses are possible. After each trial, the belief distribution is updated according to Bayes rule:

$$\begin{array}{c} P\left(h|c_{1:t},r_{1:t}\right)\propto P\left(r_{t}|h,c_{t}\right)P\left(h|c_{1:t-1},r_{1:t-1}\right). \end{array}$$ (4)

At decision time, the expected reward for each choice is calculated by marginalizing over the belief distribution:

$$\begin{array}{c} ER\left(c_{t+1}\right)=\sum_{h}P\left(h|c_{1:t},r_{1:t}\right)P\left(r_{t+1}|h,c_{t+1}\right). \end{array}$$ (5)

The expected reward is then used to determine the choice probability as in Eq 2.

We note that this model is not strictly optimal, even with no decision noise, as it maximizes the expected reward on the current trial, but not the total reward over a game.

#### Random-switch serial hypothesis testing (SHT) model

The random-switch SHT model assumes the participant tests one hypothesis at any given time. We do not directly observe what hypothesis the participant is testing, and need to infer that from their choices. We do so by using the change-point detection model in [26]. The basic idea is to infer the current hypothesis (denoted by *h*_*t*_) from all the choices the participant has made and the reward outcomes they received so far in the current game (together denoted by *d*_1:*t*−1_); see Supplementary Methods in S1 Text for implementation details. Once we obtain the posterior probability distribution over the current hypothesis *P*(*h*_*t*_|*d*_1:*t*−1_), we can use it to predict choice:

$$\begin{array}{c} P\left(c_{t}|d_{1:t-1}\right)=\sum_{h_{t}}P\left(c_{t}|h_{t}\right)P\left(h_{t}|d_{1:t-1}\right) \end{array}$$ (6)

In order to calculate *P*(*h*_*t*_|*d*_1:*t*−1_), we consider the generative model of participants’ choices. First, we determine the participant’s hypothesis space: In “known” games, participants were informed about the number of relevant dimensions, which limits the set of possible hypotheses in these games. The way people interpret and follow instructions, however, may vary. Thus, we parameterize the hypothesis space (i.e., people’s prior over all possible hypotheses) with two weight parameters *w*_*l*_ and *w*_*h*_ (before normalization):

$$\begin{array}{c} P\left(h\right)\propto \{\begin{array}{cc} w_{l} & \;\text{if}\;D(h)<D\\ 1 & \;\text{if}\;D(h)=D\\ w_{h} & \;\text{if}\;D(h)>D \end{array} \end{array}$$ (7)

Here, *D*(*h*) is the dimensionality of hypothesis *h* (how many rewarding features are in *h*), and *D* is the informed number of relevant dimensions of the current game. If a participant strictly follows the instruction, *w*_*l*_ = *w*_*h*_ = 0, i.e., only hypotheses with the same dimensionality as the instruction are considered; if the participant does not use the instruction information at all, *w*_*l*_ = *w*_*h*_ = 1, i.e., all 63 hypotheses are considered to be equally likely. For “unknown” games, the model uses the average *P*(*h*) of 1D, 2D and 3D “known” games to determine the prior probability of 1D, 2D and 3D hypotheses.

The generative model of participants’ choice behavior is assumed to contain three parts: the hypothesis-testing policy (whether to stay with the current hypothesis or switch to a new one), the hypothesis-switching policy (what the next hypothesis should be when switching hypotheses), and the choice policy given the currently tested hypothesis. The first two policies together determine the transition from the hypothesis on the previous trial to the current one, and the choice policy determines the mapping between the current hypothesis and the choice.

Following [26], we consider the following hypothesis testing policy: on each trial, the participant estimates the reward probability of the current hypothesis. Using a uniform Dirichlet prior, this is equivalent to counting how many times they have been rewarded since they started testing this hypothesis. The estimated reward probability is then compared to a soft threshold *θ* to determine whether to stay with this hypothesis or to switch to a different one:

$$\begin{array}{c} Pr\left(\text{stay}\right)=\frac{1}{1+e^{-\beta_{\text{stay}}({\hat{P}}_{\text{reward}}-\theta )}}, \end{array}$$ (8)

where ${\hat{P}}_{\text{reward}}=\frac{\text{reward}\;\text{count}\;+1}{\text{trial}\;\text{count}+2}$ is the estimated reward probability, and *β*_*stay*_ and *θ* are free parameters. If the participant decides to switch, they randomly switch to any other hypothesis according to the prior over hypotheses specified in Eq 7 (i.e. the random hypothesis-switch policy):

$$\begin{array}{c} P\left(h_{t}\right)=\{\begin{array}{c} Pr\left(\text{stay}\right),\;\text{if}\;h_{t}=h_{t-1}\\ (1-Pr(\text{stay}))\frac{P(h_{t})}{\sum_{h\neq h_{t-1}}P\left(h\right)},\;\text{if}\;h_{t}\neq h_{t-1} \end{array} \end{array}$$ (9)

Finally, we assume a choice policy where participants configure stimuli according to their hypothesis most of the time, but with a lapse rate of λ choose any configuration randomly.

#### Value-based serial hypothesis testing model

The value-based SHT model is the same as the random-switch SHT model, except that it uses a value-based hypothesis-switch policy. It maintains a set of feature values updated according to the feature RL with decay model, as in Eq 3 (but with a single learning rate), and calculates the expected reward for each alternative hypothesis by adding up its constituent feature values, similar to Eq 1 but for *h* instead of *c*. The probability of switching to *h*_*t*_ ≠ *h*_*t*−1_ is:

$$\begin{array}{c} P\left(h_{t}\right)=(1-Pr(\text{stay}))\frac{e^{\beta_{\text{switch}}\cdot ER\left(h_{t}\right)}}{\sum_{h^{\prime }\neq h_{t-1}}e^{\beta_{\text{switch}}\cdot ER\left(h^{\prime }\right)}},\; \end{array}$$ (10)

where *β*_switch_ is a free parameter.

#### Variants of the value-based SHT model

We considered several variants of the value-based SHT model by modifying the hypothesis-testing policy and the choice policy of the baseline value-based SHT model described above.

#### Not always testing a hypothesis

In the experiment, the participant could choose not to select any feature, and let the computer configure a random stimulus. Many participants did so, especially in the beginning of each game, potentially due to not having a good candidate hypothesis in mind. To model this, we add a soft threshold on hypothesis testing: if the expected reward of the best candidate hypothesis is below a threshold *θ*_test_, participants will be unlikely to test any hypothesis:

$$\begin{array}{c} Pr\left(\text{test}\right)=\frac{1}{1+e^{-\beta_{\text{test}}({\text{max}}_{h}\left(ER\left(h\right)\right)-\theta_{\text{test}})}} \end{array}$$ (11)

*β*_test_ and *θ*_test_ are additional free parameters of this model. This mechanism was applied to the first trial of each game and at hypothesis switch points.

#### Alternative hypothesis-testing policy: Using reward probability information

In the experiment, participants were informed of the reward probabilities for all game conditions (Table 1). Our baseline model did not make use of this information. One way to use such information is to consider a target reward probability *RP*_target_ for the current hypothesis *h*. If the hypothesis dimension *D*(*h*) is equal to or larger than the instructed dimension of a game (in known games) *D*, the hypothesis should attain the highest possible reward probability if all features in *h* are rewarding features so *RP*_target_ = 0.8. However, if *D*(*h*) < *D*, the target should be lower. For example, when testing the same one-dimensional hypothesis, participants should expect a higher reward probability if they are in a 1D game (*RP*_target_ = 0.8) compared to in a 3D game (*RP*_target_ = 0.4). In “known” games, we therefore assumed that participants set their thresholds *θ* for switching hypotheses according to this target reward probability, with a free-parameter offset *δ*:

$$\begin{array}{c} \theta =RP_{\text{target}}+\delta \end{array}$$ (12)

For “unknown” games, we assume participants use the average *RP*_target_ of 1D, 2D and 3D “known” games, such that *RP*_target_ = 0.6, 0.733 and 0.8 for 1D, 2D and 3D hypotheses, respectively.

#### Alternative choice policy: Selecting more features than prescribed by the hypothesis

In the baseline model, participants’ choices are assumed to be aligned with their current hypothesis, unless they lapse in their choice. In the experiment, however, we observed an overall tendency to select more features than instructed (Fig 2B). This was not surprising as there was no cost to selecting more features. In fact, it was strictly optimal to always make selections on all dimensions, as there was always a best feature within each dimension (at least equally good as the other two), and holding all features fixed helps test the current hypothesis (the computer randomly chooses features for any unselected dimensions, meaning that reward attained could be due to those features and not the hypothesis tested). Thus, we assumed in this alternative model that participants may select more features than their current hypothesis *h*_*t*_. The probability for choices that are supersets of *h*_*t*_ was determined by the difference in the numbers of dimensions compared to *h*_*t*_, with a decay rate *k* as a free parameter:

$$\begin{array}{c} P\left(c_{t}|h_{t}\right)\propto e^{k(D\left(c_{t}\right)-D\left(h_{t}\right))} \end{array}$$ (13)

In this model, participants could still lapse, meaning that all choices that are not supersets of *h*_*t*_ were equally likely, with probabilities that summed to λ.

### Model fitting and model comparison

We fit the models to each participant’s data using maximum likelihood estimation. We used the minimize function (L-BFGS-B algorithm) in Python package scipy.optimize as the optimizer; each optimization was repeated 10 times with random starting points. Models were evaluated with leave-one-game-out cross-validation: the likelihood of each game was calculated using the parameters obtained by fitting the other 17 games; the geometric average likelihood per trial across all held-out games is reported (i.e., total log likelihood across all trials a participant played divided by number of trials and exponentiated, and then averaged over participants).

## Supporting information

S1 Fig Additional behavioral results. (A, B) Same as Fig 2A and 2B but aggregated by known v.s. unknown games. (C) Post-game responses to questions about the rewarding features in each game condition. Kwn = known games, Unk = unknown games. After each game, participants were asked to report the rewarding feature for each dimension, or indicate this dimension as irrelevant to reward. Responses are classified into five categories. Correct feature: correctly identifying a rewarding feature; Incorrect feature: incorrectly reporting a non-rewarding feature as rewarding for a relevant dimension; Miss relevance: reporting a relevant dimension as irrelevant; False positive: incorrectly reporting a rewarding feature for an irrelevant dimension; Correct rejection: correctly identifying an irrelevant dimension. (D, E, F) The type of feature selection, the number of features changed in choices, and the type of choice change as a function of trial index, broken down by game types. (D) The number of features selected by participants was broken down into three types: correct, incorrect or false positive (i.e. selecting a feature when that dimension was irrelevant), and summed across three dimensions. Over the game, the number of correct features increased and the number of incorrect features decreased, consistent across all game types and indicating learning. The trends were mostly consistent between known and unknown games, except for 1D games: false positive responses decreased in the known condition but stayed steady in the unknown condition. These results are consistent with post-game questions (Fig 2D; participants were more likely to make false-positive responses in 1D unknown games compared to 1D known games). Interestingly, when games were more complex (e.g., 2D games), participants were unable to reduce false positive responses over time even in the known condition. (E) The average number of features changed from one choice to the next, for all trials (upper panel) and only for trials with a choice change (lower panel). Overall, participants changed more features in their choice in the beginning of a game, and this decreased over time. The pattern was mostly consistent across game types, except for 1D games: the reduction was slower in the known condition compared to the unknown condition. Specifically, in 1D known games, participants continued to change their choices in the later part of the game, despite already obtaining a high reward rate, suggesting that they were trying to further narrow down and find the exact rewarding feature, potentially driven by the game instruction (one dimension was relevant). This is consistent with a lower false-positive rate in 1D known games compared to 1D unknown games. In 3D games, this pattern is reversed, likely because participants knew there was no need to narrow down in 3D known games after achieving the maximal reward rate. (F) Choice change was divided into five categories: adding features (e.g. red to red circle), dropping features (e.g. red circle to red), switching within dimension (e.g. red circle to blue circle), switching across dimensions (e.g. red to circle), and all other changes (any mixture of the previous four types, e.g. red circle to blue). Among the five types, switching within dimension was the most common. There were very few occurrences of the mixture type (“Others”); whereas for a random-choice policy, this would be the most common type. This suggests that participants tended to make local, systematical changes in their choices, further supporting a serial hypothesis testing process.
(PDF)

S2 Fig Additional model fitting results. (A) Model fits broken down for each game type. (B) The fitted prior probability for 1/2/3D hypothesis (x-axis) in different game types (subplots) in the main value-based SHT model. In known games, participants had a higher prior probability for the hypotheses consistent with the task instructions (darker red bars). In unknown games, more complex hypotheses were deemed *a priori* more likely.
(PDF)

S3 Fig Learning curves for data and all model simulations. Top and fourth rows are identical to Figs 2A, 2B, 4B and 4C, respectively.
(PDF)

S4 Fig Variants of the serial hypothesis-testing (SHT) model. (A) A diagram of the SHT models compared in the main text. Different variants for each model assumption are presented in colored boxes: in gray are the assumptions adopted by the baseline model; colors denote the different variants tested. (B) Difference in average likelihood per trial between variants of the SHT models and the baseline value-based SHT model. Each model except the full model is only different from the baseline model by one assumption as noted in the label; the full model adopts the better alternative in every assumption. Bar colors correspond to those in panel A, except for the full model (in white). Specifically, the purple bar corresponds to the random-switch SHT model. Error bars represent ±1 s.e.m. across participants.
(PDF)

S1 Text Inference in the serial hypothesis-testing models. (PDF)
