# Correction: Humans combine value learning and hypothesis testing strategically in multi-dimensional probabilistic reward learning

**DOI:** 10.1371/journal.pcbi.1010775

**Published:** 2022-12-13

**Authors:** The PLOS Computational Biology Staff

The Funding information for this article is incomplete. The correct Funding statement is:

This work was supported by Grant R01DA042065 from the National Institute of Drug Abuse (YN), Grant W911NF-14-1-0101 from the Army Research Office (YN), and the World Premier International Research Center Initiative (WPI), MEXT, Japan (MBC). The funders had no role in study design, data collection and analysis, decision to publish, or preparation of the manuscript.
