# Supplementary material for: Humans combine value learning and hypothesis testing strategically in multi-dimensional probabilistic reward learning
Source: PLoS Comput Biol. 2022 Nov 23;18(11):e1010699. doi: 10.1371/journal.pcbi.1010699 (PMC9683628; doi:10.1371/journal.pcbi.1010699)
Supplement: S1 Fig — (A, B) Same as Fig 2A and 2B but aggregated by known v.s. unknown games. (C) Post-game responses to questions about the rewarding features in each game condition. Kwn = known games, Unk = unknown games. After each game, participants were asked to report the rewarding feature for each dimension, or indicate this dimension as irrelevant to reward. Responses are classified into five categories. Correct feature: correctly identifying a rewarding feature; Incorrect feature: incorrectly reporting a non-rewarding feature as rewarding for a relevant dimension; Miss relevance: reporting a relevant dimension as irrelevant; False positive: incorrectly reporting a rewarding feature for an irrelevant dimension; Correct rejection: correctly identifying an irrelevant dimension. (D, E, F) The type of feature selection, the number of features changed in choices, and the type of choice change as a function of trial index, broken down by game types. (D) The number of features selected by participants was broken down into three types: correct, incorrect or false positive (i.e. selecting a feature when that dimension was irrelevant), and summed across three dimensions. Over the game, the number of correct features increased and the number of incorrect features decreased, consistent across all game types and indicating learning. The trends were mostly consistent between known and unknown games, except for 1D games: false positive responses decreased in the known condition but stayed steady in the unknown condition. These results are consistent with post-game questions (Fig 2D; participants were more likely to make false-positive responses in 1D unknown games compared to 1D known games). Interestingly, when games were more complex (e.g., 2D games), participants were unable to reduce false positive responses over time even in the known condition. (E) The average number of features changed from one choice to the next, for all trials (upper panel) and only for trials with a choice change (lo [file pcbi.1010699.s001.pdf]

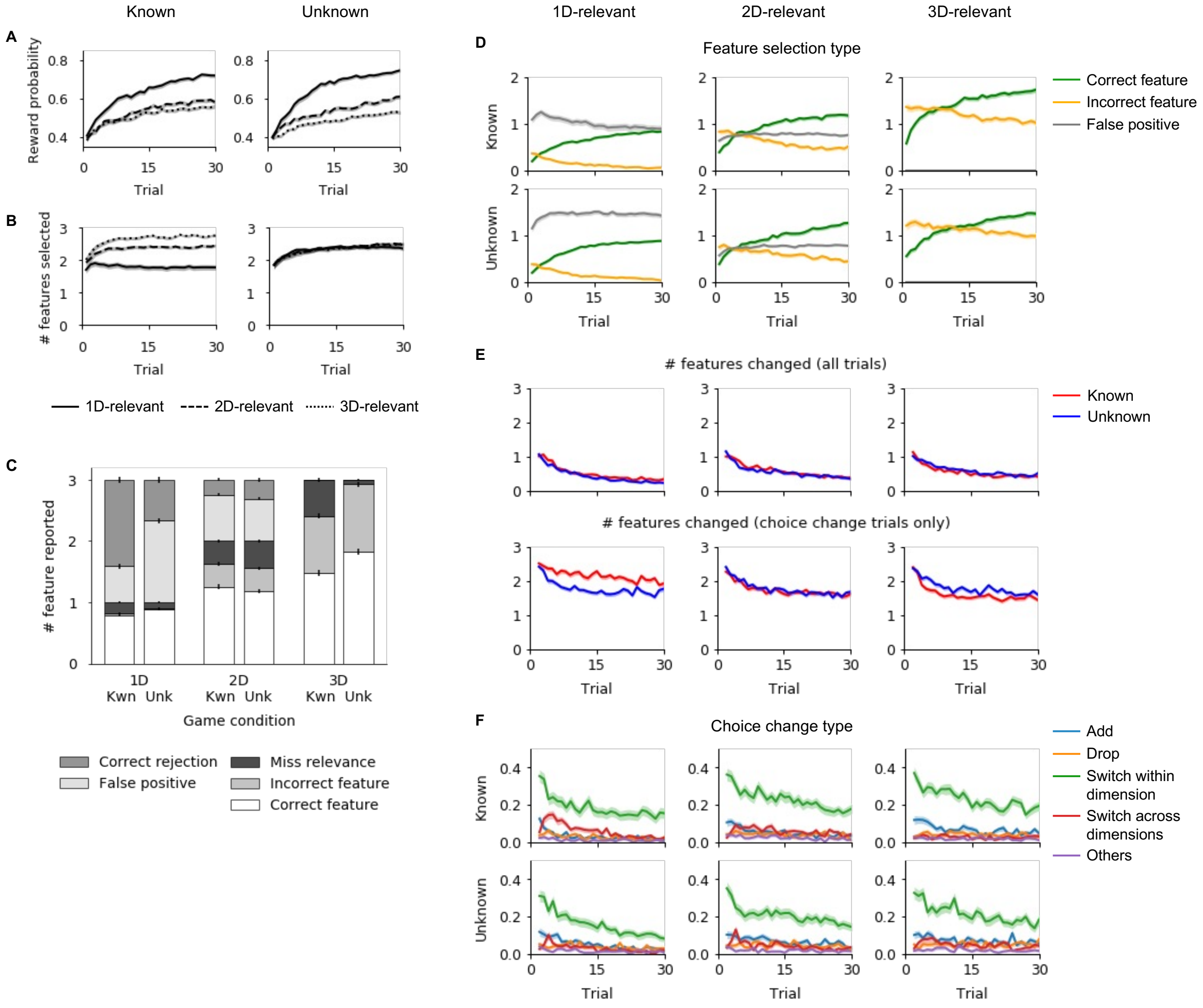

S1 Fig: Additional behavioral results. (A, B) Same as Fig 2A, 2B but aggregated by known v.s. unknown games. (C) Post-game responses to questions about the rewarding features in each game condition. Kwn = known games, Unk = unknown games. After each game, participants were asked to report the rewarding feature for each dimension, or indicate this dimension as irrelevant to reward. Responses are classified into five categories. Correct feature: correctly identifying a rewarding feature; Incorrect feature: incorrectly reporting a non-rewarding feature as rewarding for a relevant dimension; Miss relevance: reporting a relevant dimension as irrelevant; False positive: incorrectly reporting a rewarding feature for an irrelevant dimension; Correct rejection: correctly identifying an irrelevant dimension. **(D, E, F) The type of feature selection, the number of features changed in choices, and the type of choice change as a function of trial index, broken down by game types.** (D) The number of features selected by participants was broken down into three types: correct, incorrect or false positive (i.e. selecting a feature when that dimension was irrelevant), and summed across three dimensions. Over the game, the number of correct features increased and the number of incorrect features decreased, consistent across all game types and indicating learning. The trends were mostly consistent between known and unknown games, except for 1D games: false positive responses decreased in the known condition but stayed steady in the unknown condition. These results are consistent with post-game questions (Fig 2D; participants were more likely to make false-positive responses in 1D unknown games compared to 1D known games). Interestingly, when games were more complex (e.g., 2D games), participants were unable to reduce false positive responses over time even in the known condition. (E) The average number of features changed from one choice to the

next, for all trials (upper panel) and only for trials with a choice change (lower panel). Overall, participants changed more features in their choice in the beginning of a game, and this decreased over time. The pattern was mostly consistent across game types, except for 1D games: the reduction was slower in the known condition compared to the unknown condition. Specifically, in 1D known games, participants continued to change their choices in the later part of the game, despite already obtaining a high reward rate, suggesting that they were trying to further narrow down and find the exact rewarding feature, potentially driven by the game instruction (one dimension was relevant). This is consistent with a lower false-positive rate in 1D known games compared to 1D unknown games. In 3D games, this pattern is reversed, likely because participants knew there was no need to narrow down in 3D known games after achieving the maximal reward rate. (F) Choice change was divided into five categories: adding features (e.g. red to red circle), dropping features (e.g. red circle to red), switching within dimension (e.g. red circle to blue circle), switching across dimensions (e.g. red to circle), and all other changes (any mixture of the previous four types, e.g. red circle to blue). Among the five types, switching within dimension was the most common. There were very few occurrences of the mixture type ("Others"); whereas for a random-choice policy, this would be the most common type. This suggests that participants tended to make local, systematical changes in their choices, further supporting a serial hypothesis testing process.
