# Supplementary material for: Humans combine value learning and hypothesis testing strategically in multi-dimensional probabilistic reward learning
Source: PLoS Comput Biol. 2022 Nov 23;18(11):e1010699. doi: 10.1371/journal.pcbi.1010699 (PMC9683628; doi:10.1371/journal.pcbi.1010699)
Supplement: S2 Fig — (A) Model fits broken down for each game type. (B) The fitted prior probability for 1/2/3D hypothesis (x-axis) in different game types (subplots) in the main value-based SHT model. In known games, participants had a higher prior probability for the hypotheses consistent with the task instructions (darker red bars). In unknown games, more complex hypotheses were deemed a priori more likely. (PDF) [file pcbi.1010699.s002.pdf]

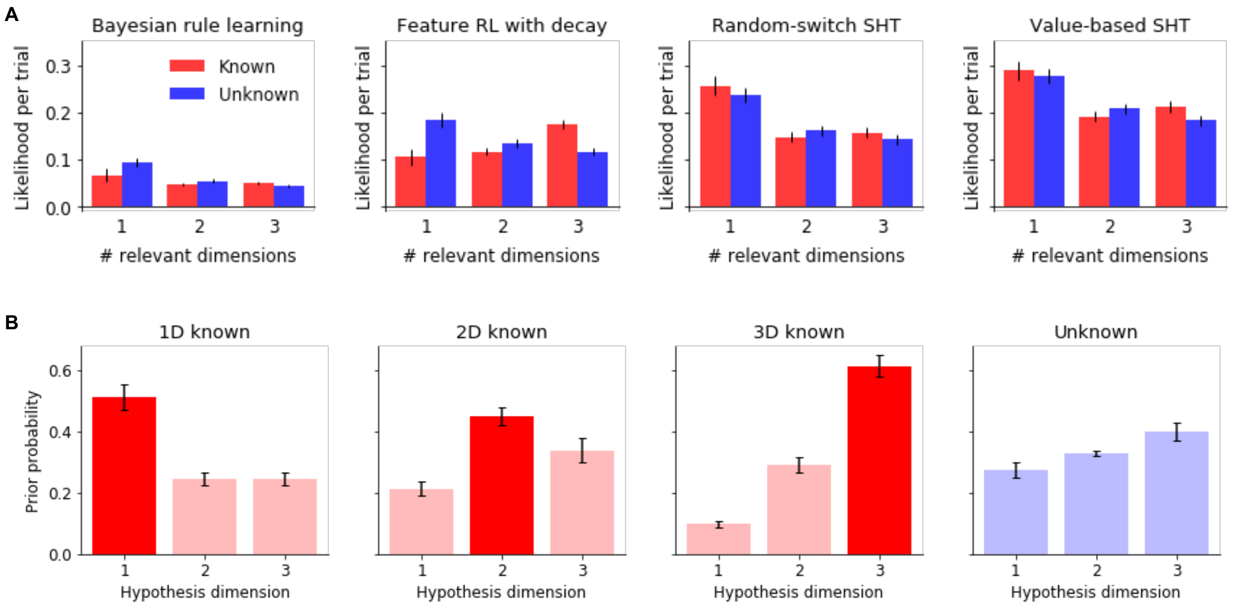


**S2 Fig: Additional model fitting results. (A)** Model fits broken down for each game type. **(B)** The fitted prior probability for 1/2/3D hypothesis (x-axis) in different game types (subplots) in the main value-based SHT model. In known games, participants had a higher prior probability for the hypotheses consistent with the task instructions (darker red bars). In unknown games, more complex hypotheses were deemed *a priori* more likely.
