# Supplementary material for: Humans combine value learning and hypothesis testing strategically in multi-dimensional probabilistic reward learning
Source: PLoS Comput Biol. 2022 Nov 23;18(11):e1010699. doi: 10.1371/journal.pcbi.1010699 (PMC9683628; doi:10.1371/journal.pcbi.1010699)
Supplement: S3 Fig — Top and fourth rows are identical to Figs 2A, 2B, 4B and 4C, respectively. (PDF) [file pcbi.1010699.s003.pdf]

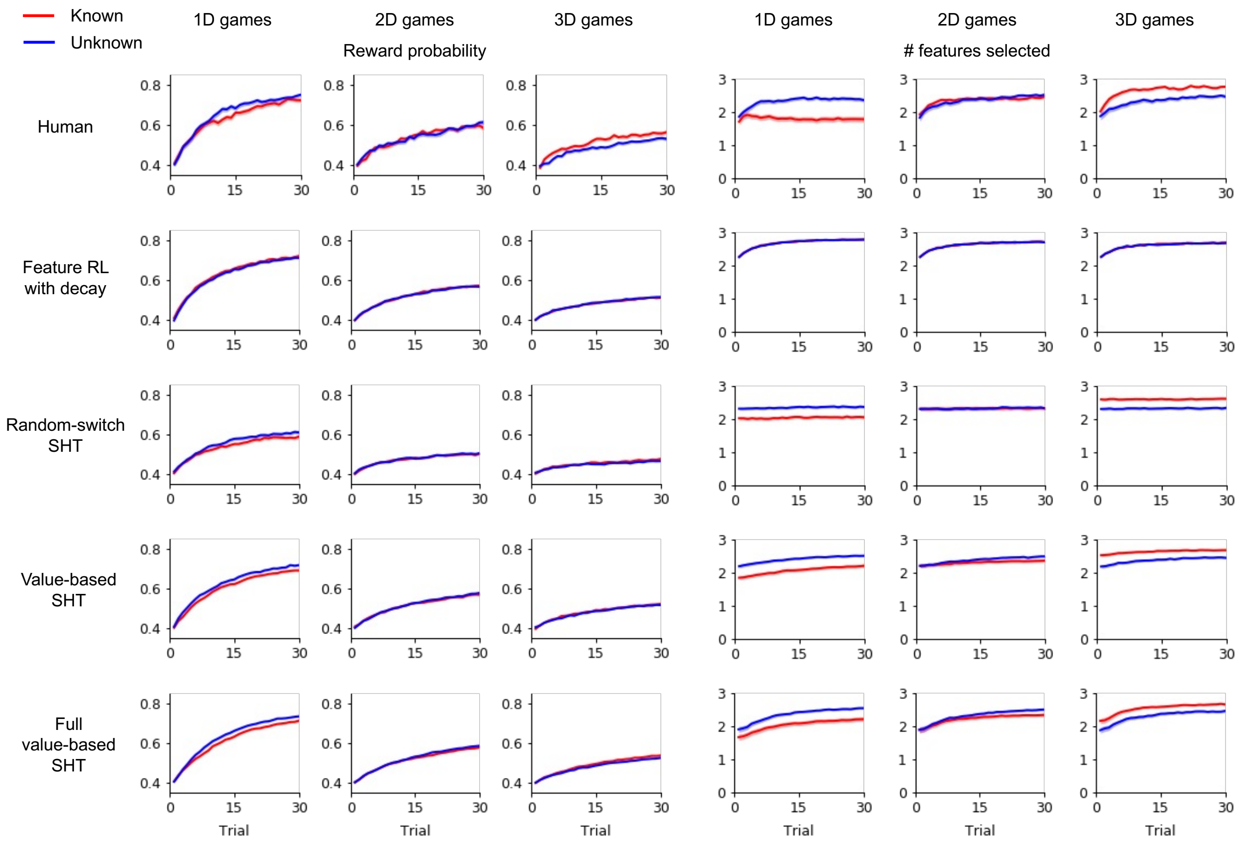

S3 Fig: Learning curves for data and all model simulations. Top and fourth rows are identical to Fig 2A,B and Fig 4B,C, respectively.
