# Supplementary material for: Humans combine value learning and hypothesis testing strategically in multi-dimensional probabilistic reward learning
Source: PLoS Comput Biol. 2022 Nov 23;18(11):e1010699. doi: 10.1371/journal.pcbi.1010699 (PMC9683628; doi:10.1371/journal.pcbi.1010699)
Supplement: S4 Fig — (A) A diagram of the SHT models compared in the main text. Different variants for each model assumption are presented in colored boxes: in gray are the assumptions adopted by the baseline model; colors denote the different variants tested. (B) Difference in average likelihood per trial between variants of the SHT models and the baseline value-based SHT model. Each model except the full model is only different from the baseline model by one assumption as noted in the label; the full model adopts the better alternative in every assumption. Bar colors correspond to those in panel A, except for the full model (in white). Specifically, the purple bar corresponds to the random-switch SHT model. Error bars represent ±1 s.e.m. across participants. (PDF) [file pcbi.1010699.s004.pdf]

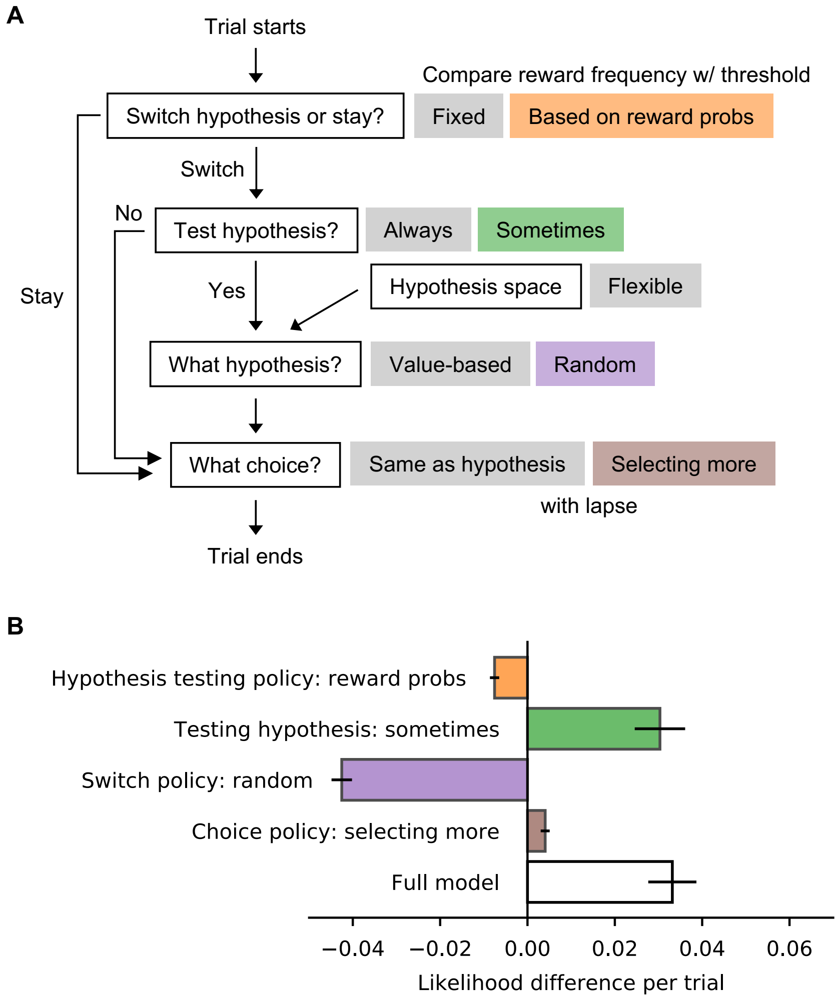

S4 Fig: Variants of the serial hypothesis-testing (SHT) model. (A) A diagram of the SHT models compared in the main text. Different variants for each model assumption are presented in colored boxes: in gray are the assumptions adopted by the baseline model; colors denote the different variants tested. **(B)** Difference in average likelihood per trial between variants of the SHT models and the baseline value-based SHT model. Each model except the full model is only different from the baseline model by one assumption as noted in the label; the full model adopts the better alternative in every assumption. Bar colors correspond to those in panel A, except for the full model (in white). Specifically, the purple bar corresponds to the random-switch SHT model. Error bars represent ± 1 s.e.m. across participants.
