# Supplementary material for: Humans combine value learning and hypothesis testing strategically in multi-dimensional probabilistic reward learning
Source: PLoS Comput Biol. 2022 Nov 23;18(11):e1010699. doi: 10.1371/journal.pcbi.1010699 (PMC9683628; doi:10.1371/journal.pcbi.1010699)
Supplement: S1 Text — (PDF) [file pcbi.1010699.s005.pdf]

S1 Supplementary Methods: Inference in the serial hypothesis-testing models

The random-switch and value-based SHT models assume that participants test hypotheses serially. As experimenters, however, we do not observe what hypotheses they are testing. In order to predict their choice c_t on trial t, we need to marginalize over all possible hypotheses h_t:

$$P(c_t|d_{1:t-1}) = \sum_{h_t} P(c_t|h_t) P(h_t|d_{1:t-1})$$

The first term $P(c_t|h_t)$ is given by the choice policy, as discussed before. In this section, we describe how we calculated the second term, i.e., how we inferred the hypothesis that the participant is currently testing based on their choice and reward history ($d_{1:t-1}$). We do so using the change point detection model in [1]. According to this model, only choice history is relevant to inference, i.e., $d_{1:t-1} = c_{1:t-1}$. Therefore, $P(h_t|d_{1:t-1}) = P(h_t|c_{1:t-1})$.

We first introduce the run-length of hypothesis on trial t as l_t, i.e., how long the participant has been testing the current hypothesis. The probability of the current hypothesis can then be written as the marginalization over run-length of the current and previous trials (on the first trial, all hypotheses are equally likely, corresponding to a uniform prior on h_1):

$$\begin{aligned}
P(h_t|c_{1:t-1}) &= \sum_{l_t, l_{t-1}} P(h_t|l_t, l_{t-1}, c_{1:t-1}) P(l_t, l_{t-1}|c_{1:t-1}) \\
&= \sum_{l_t, l_{t-1}} P(h_t|l_t, l_{t-1}, c_{1:t-1}) P(l_t|l_{t-1}, c_{1:t-1}) P(l_{t-1}|c_{1:t-1}) \\
&= \sum_{l_t, l_{t-1}} \sum_{h_{t-1}} \left(P(h_t|l_t, h_{t-1}, l_{t-1}, c_{1:t-1}) P(h_{t-1}|l_{t-1}, c_{1:t-1}) \right) P(l_t|l_{t-1}, c_{1:t-1}) P(l_{t-1}|c_{1:t-1})
\end{aligned}$$

For the rest of this section, we describe how to calculate each (color-coded) term.

The second term $P(h_{t-1}|l_{t-1}, c_{1:t-1})$: recursive calculation

The second term can be calculated recursively using Bayes rule (normalization is needed):

$$P(h_{t-1}|l_{t-1}, c_{1:t-1}) \propto P(c_{t-1}|h_{t-1}, l_{t-1}, c_{1:t-2}) P(h_{t-1}|l_{t-1}, c_{1:t-2})$$

On the second trial (special case with $t = 2$), $P(h_1|l_1, d_1) \propto P(c_1|h_1) P(h_1|l_1)$, where $P(h_1|l_1)$ is set to the prior belief distribution (uniform distribution). Then, from the third trial:

$$\begin{aligned}
P(h_{t-1}|l_{t-1}, c_{1:t-1}) &\propto P(c_{t-1}|h_{t-1}) P(h_{t-1}|l_{t-1}, c_{1:t-2}) \\
&= P(c_{t-1}|h_{t-1}) \sum_{l_{t-2}} P(h_{t-1}|l_{t-1}, l_{t-2}, c_{1:t-2}) P(l_{t-2}|c_{1:t-2}) \\
&= P(c_{t-1}|h_{t-1}) \sum_{l_{t-2}} \sum_{h_{t-2}} \left(P(h_{t-1}|l_{t-1}, h_{t-2}, l_{t-2}, c_{1:t-2}) P(h_{t-2}|l_{t-2}, c_{1:t-2}) \right) P(l_{t-2}|c_{1:t-2})
\end{aligned}$$

where $P(c_{t-1}|h_{t-1})$ is given by the choice policy.

The fourth term $P(l_{t-1}|c_{1:t-1})$: recursive calculation

The fourth term is initialized as an array of a single element 1 on the second trial, and can be calculated recursively using Bayes rule starting from the third trial:

$$
\begin{aligned}
P(l_{t-1}|c_{1:t-1}) \propto & P(c_{t-1}|l_{t-1}, c_{1:t-2})P(l_{t-1}|c_{1:t-2}) \\
= & \sum_{h_{t-1}} P(c_{t-1}|h_{t-1}, l_{t-1}, c_{1:t-2})P(h_{t-1}|l_{t-1}, c_{1:t-2}) \sum_{l_{t-2}} P(l_{t-1}|l_{t-2}, c_{1:t-2})P(l_{t-2}|c_{1:t-2}) \\
= & \sum_{h_{t-1}} P(c_{t-1}|h_{t-1}) \sum_{l_{t-2}} P(h_{t-1}|l_{t-1}, l_{t-2}, c_{1:t-2})P(l_{t-2}|c_{1:t-2}) \sum_{l_{t-2}} P(l_{t-1}|l_{t-2}, c_{1:t-2})P(l_{t-2}|c_{1:t-2}) \\
= & \sum_{h_{t-1}} P(c_{t-1}|h_{t-1}) \sum_{l_{t-2}} \sum_{h_{t-2}} \left(P(h_{t-1}|l_{t-1}, h_{t-2}, l_{t-2}, c_{1:t-2})P(h_{t-2}|l_{t-2}, c_{1:t-2})\right) P(l_{t-2}|c_{1:t-2}) \\
& \sum_{l_{t-2}} P(l_{t-1}|l_{t-2}, c_{1:t-2})P(l_{t-2}|c_{1:t-2})
\end{aligned}
$$

The third term $P(l_t|l_{t-1}, c_{1:t-1})$: hypothesis-testing policy

We calculated the third term $P(l_t|l_{t-1}, c_{1:t-1})$ by marginalizing over the hypothesis from the previous trial:

$$P(l_t|l_{t-1}, c_{1:t-1}) = \sum_{h_{t-1}} P(l_t|h_{t-1}, l_{t-1}, c_{1:t-1})P(h_{t-1}|l_{t-1}, c_{1:t-1})$$

The serial hypothesis-testing assumption implies that l_t can only take on two possible values: $l_{t-1}+1$ if the participant stayed with the hypothesis from last trial, or 0 if they switched hypotheses.

$$
\begin{aligned}
P(l_t = l_{t-1} + 1|h_{t-1}, l_{t-1}, c_{1:t-1}) &= P_{\text{stay}} \\
P(l_t = 0|h_{t-1}, l_{t-1}, c_{1:t-1}) &= 1 - P_{\text{stay}}
\end{aligned}
$$

P_{stay} is a function of $h_{t-1}, l_{t-1}, c_{1:t-1}$, and is determined by the participant's hypothesis-testing policy (Equation **??**).

The first term $P(h_t|l_t, h_{t-1}, l_{t-1}, c_{1:t-1})$: hypothesis-switch policy

The first term $P(h_t|l_t, h_{t-1}, l_{t-1}, c_{1:t-1})$ is given by the switch policy. As noted before, only certain combinations of l_t and l_t values are possible: either $l_t = l_{t-1} + 1$ (stay), or $l_t = 0$ (switch). Specifically, for $l_t = l_{t-1} + 1$ (stay), $P(h_t = h_{t-1}|h_{t-1}, l_t, l_{t-1}, c_{1:t-1}) = 1$, otherwise, 0; for $l_t = 0$ (switch), $P(h_t \neq h_{t-1}|h_{t-1}, l_t, l_{t-1}, c_{1:t-1})$ is determined as in Equations **??** and **??**. Other l_t, l_{t-1} combinations all have probability zero.

References

1. Wilson RC, Niv Y. Inferring relevance in a changing world. Frontiers in human neuroscience. 2012;5:189.
